# When it comes to genomic analysis of tumours, don't buy in bulk

**DOI:** 10.1038/s41416-018-0096-8

**Published:** 2018-05-10

**Authors:** Jorge Nieva, James Hicks

**Affiliations:** 1 USC/Norris Cancer Center, Los Angeles, CA 90089 USA; 2 Michelson Center for Convergent Biosciences, USC Dornsife College of Letters, Arts & Sciences and USC/Norris Cancer Center, Los Angeles, CA 90089 USA

## Abstract

In a recent publication, single-cell transcription analysis was coupled with histology and cell biology to allow revision of the squamous head and neck cancer (HNSCC) subtypes. The study revealed the presence, location and function of novel tumour cell phenotypes related to metastasis in HNSCC.

## Main

For any molecular classification system to be adopted by clinicians, there must be treatment implications. Hence, when breast cancer was classified by gene expression into *basaloid*, *Her2* and *luminal* types,^1^ it was rapidly adopted, because each type had an implication for drug therapy. In a recent edition of *Cell*, Puram et al.^2^ have investigated the cellular complexity of squamous head and neck cancer (HNSCC) cells by examining their genomic diversity, much as a naturalist would explore the species diversity within a new genus. Classification of head and neck cancers has so far been based on The Cancer Genome Atlas (TCGA), which used bulk analysis of tumour cells to classify these cancers into classical, atypical, basal and mesenchymal groupings;^3^ this scheme also recognised the favourable prognosis of the atypical designation associated with human papillomavirus (HPV) infection.^4^ Traditional molecular analyses of resected tumour specimens are complicated by the variety of malignant cells and non-malignant stromal cells that comprise the tumour tissue. However, rather than describing the average genomic signal from the bulk analysis of a homogenised tumour, Puram et al.^2^ performed single-cell RNA sequencing on 6000 individual cells from 18 patients to explore the diversity of cells within the population, literally creating a molecular and cellular atlas of the disease.

Key to this single-cell study was the use of complementary genomic techniques, including copy-number profiling and expression phenotyping. The aim of this approach was to distinguish individual malignant cells with aneuploid genomes from the genomically normal, but phenotypically distinct stromal populations, including cancer-associated fibroblasts and immunocytes. Analysis of the expression profiles revealed that the malignant cells fell into separate clusters according to cancer subtype, whereas the stromal cells from all cases clustered together around identifiable cell phenotypes. This very basic separation of the genomically altered malignant cells, followed by further classification of the stromal subpopulations, made it possible to reach conclusions that would not have been possible using bulk tumour tissue, and sets a precedent for future single-cell tumour studies.

A great deal can be learned by taking this approach to the study of actual patient samples. The key therapeutic decisions that must be made for any patient with HNSCC are whether chemotherapy and radiation should be added to surgery. The most powerful predictors for these decisions are the presence of tumour cells outside of the lymph node capsule, and the inability to clear the surgical margin of tumour; both are surrogates for invasiveness. Additional clinical factors that guide decision-making include tumour grade and HPV infection, and the tracking of tumour cells along blood vessels and nerves. This introduces the first key finding from this single-cell analysis: the four HNSCC classifications derived from bulk analysis samples in TCGA^3^ can actually be reduced to three (malignant-basal, classical and atypical). The authors convincingly demonstrate that the previously recognised mesenchymal subtype is an artefact of bulk gene expression analysis, where the signatures of the underlying basal-like malignant cells were overwhelmed by a large stromal component, leading to the false attribution of a mesenchymal phenotype to the malignant cells. This study therefore divorces the therapeutic choices from the bulk genomic signature of the tumour, and thus simplifies the molecular classification of HNSCC from the clinical markers of invasiveness through removal of the mesenchymal subtype. This is a key finding, because microanatomy and HPV positivity currently remain more important than molecular classification in determining therapy. Furthermore, the recognition of this false attribution also creates a new set of questions: what makes a basal cancer develop a stromal component if the genetics of the tumour cells themselves are not the determinate? Is it simply because those tumours with stromal invasion were the ones that had more time to interact with the host? Stromal invasion is associated with worse prognosis and several key prognostic factors, such as extracapsular extension and lymphovascular invasion,^5^ all of which could be influenced by time, host factors (such as cytokines) or anatomic factors outside of the tumour. Pieces of the puzzle that remained stubbornly obscure in previous studies.

The next key finding bears directly on the topic of stromal invasion. The purity of the cell types achieved by single-cell analysis enabled the authors to approach a mechanistic view of the host factors and anatomical features involved in stromal invasion and, by inference, metastasis. Through expression profiling, they identified a specific phenotype in the malignant-basal cells of the most aggressive subtype, a version of the epithelial–mesenchymal transition called ‘partial EMT’ (p-EMT). p-EMT is associated with acquired cell mobility and thus increased metastatic potential.^6, 7^ This phenotype had not been detected in previous bulk studies of head and neck cancer using the TCGA collection. Using the molecular p-EMT signature, the authors were able to revisit histology samples and locate the p-EMT cells at the ‘leading edge’ of the tumour, where they interact with stromal cells, and further, they were able to detect several of the critical signalling pathways in action at that interface.

This capability to identify a cell’s expression state and subsequently locate that cell type within the macrostructure of the tumour using histology is very powerful, and greatly amplifies the information that can be derived from the single-cell analysis of a dissociated cell population. The authors also show that atypical HNSCC tumours have the lowest p-EMT score and are thus deserving of a different clinical approach to care, with reduced intensity of adjuvant treatment.^8^ HPV positive tumours of the head and neck more frequently exhibit the atypical gene expression pattern; therefore, these observations raise the question of whether HPV status is the best surrogate for defining the best prognosis tumours, or whether something like the p-EMT score would not be equally informative. The finding that only around half of the atypical tumours in the TCGA set were HPV positive^3^ also raises the question of what other source of genomic injury could lead to the same pattern of gene expression, if it is not HPV. There is a great opportunity here for follow-on work using this technique to understand the biology of atypical-type HNSCC in the HPV positive and negative settings. Of relevance, the American Joint Commission on Cancer (AJCC) will this year begin to assess HPV+ tumours of the head and neck with a different staging system than is applied to other cancers.^9^ In the new Version 8 of the staging system, nodal involvement does not upstage HPV+ oropharynx cancer patients until there are more than four lymph nodes involved, thus reflecting a better prognosis for atypical tumours even when there has been greater spread of disease.

Finally, as others have shown,^10^ this manuscript confirms that the study of HNSCC and cancer in general must come from patient samples and not from cell lines. The authors demonstrate that a variety of head and neck cancer cell lines are more related to each other than they are to any of the subtypes of the actual human disease they represent, based on their global gene expression patterns. It is becoming apparent that this disconnect between cancer models and actual tumours likely holds across most cancer types.^11^ Further, it is clear from this study that critical features of cell survival and prognosis for the HNSCC subtypes are encoded within the hypoxia and stress phenotypes as well as the novel EMT state, and each of these factors has a different pattern within the microanatomy of the patient. Together, these observations add to the urgency of focusing on actual tumours rather than models.

The clinical benefit of this type of analysis is clearly evident. The recognition that markers of phenotypic plasticity go hand-in-hand with a worse prognosis^12^ reinforces the fact that the most powerful tool we currently have to treat HNSCC is surgery, a modality that reduces the cancer cell population size without any kind of selection on the basis of cellular phenotype or diversity. Within any ecosystem, the degree of diversity within a population is a favourable trait that improves the overall fitness of a population; although the authors do not provide any kind of global diversity score for the population under study, such a score could serve as a guide for determining which tumours have the highest risk of evolving drug resistance. It should be noted that the cancers in which DNA damaging therapy with radiation and chemotherapy is sufficient, and surgery is currently optional, are those that come from the HPV+ atypical grouping, which the authors note has a lower frequency of phenotypic plasticity than the malignant-basal type.

Ultimately, if we are ever going to cure, or even manage, the incredible range of human cancers in all their complexity, we must at some point make the effort to deconstruct and reverse-engineer a variety of tumour types at the cellular and molecular level. Resected tumours could be likened to captured enemy spacecraft, to be disassembled, piece by piece, until we understand their weaponry, propulsion and defence systems, and ultimately, their vulnerability. While single-cell molecular analysis is time-, effort- and money-intensive, it is, as the Puram study shows, a powerful tool for studying the blueprints of a cancer. Whereas several key clinical questions remain unanswered for the moment, such as how expression levels of the EMT, stress and hypoxia programs or the three subtypes of HNSCC affect response to drug therapy, without exposing this part of the ‘alien blueprint’, they would not even be asked.
